# Relationships between IgE/IgG_4_ Epitopes, Structure and Function in *Anisakis simplex* Ani s 5, a Member of the SXP/RAL-2 Protein Family

**DOI:** 10.1371/journal.pntd.0002735

**Published:** 2014-03-06

**Authors:** María Flor García-Mayoral, Miguel Angel Treviño, Teresa Pérez-Piñar, María Luisa Caballero, Tobias Knaute, Ana Umpierrez, Marta Bruix, Rosa Rodríguez-Pérez

**Affiliations:** 1 Institute of Physical Chemistry “Rocasolano”. CSIC. Madrid, Spain; 2 Carlos III Hospital, Immunology Department, Madrid, Spain; 3 JPT Peptide Technologies, Berlin, Germany; 4 Carlos III Hospital, Allergy Department, Madrid, Spain; James Cook University, Australia

## Abstract

**Background:**

Anisakiasis is a re-emerging global disease caused by consumption of raw or lightly cooked fish contaminated with L3 *Anisakis* larvae. This zoonotic disease is characterized by severe gastrointestinal and/or allergic symptoms which may misdiagnosed as appendicitis, gastric ulcer or other food allergies.

The *Anisakis* allergen Ani s 5 is a protein belonging to the SXP/RAL-2 family; it is detected exclusively in nematodes. Previous studies showed that SXP/RAL-2 proteins are active antigens; however, their structure and function remain unknown.

The aim of this study was to elucidate the three-dimensional structure of Ani s 5 and its main IgE and IgG_4_ binding regions.

**Methodology/Principal Findings:**

The tertiary structure of recombinant Ani s 5 in solution was solved by nuclear magnetic resonance. Mg^2+^, but not Ca^2+^, binding was determined by band shift using SDS-PAGE. IgE and IgG_4_ epitopes were elucidated by microarray immunoassay and SPOTs membranes using sera from nine *Anisakis* allergic patients.

The tertiary structure of Ani s 5 is composed of six alpha helices (H), with a Calmodulin like fold. H3 is a long, central helix that organizes the structure, with H1 and H2 packing at its N-terminus and H4 and H5 packing at its C-terminus. The orientation of H6 is undefined. Regarding epitopes recognized by IgE and IgG4 immunoglobulins, the same eleven peptides derived from Ani s 5 were bound by both IgE and IgG_4_. Peptides 14 (L40-K59), 26 (A76-A95) and 35 (I103-D122) were recognized by three out of nine sera.

**Conclusions/Significance:**

This is the first reported 3D structure of an *Anisakis* allergen. Magnesium ion binding and structural resemblance to Calmodulin, suggest some putative functions for SXP/RAL-2 proteins. Furthermore, the IgE/IgG_4_ binding regions of Ani s 5 were identified as segments localized on its surface. These data will contribute towards a better understanding of the interactions that occur between immunoglobulins and allergens and, in turn, facilitate the design of novel diagnostic tests and immunotherapeutic strategies.

## Author Summary

Knowledge of potential pathogens in seafood is of major significance for human health. The high rates of parasitation of fish all over the world make *Anisakis* a serious health hazard. In fact, Anisakiasis is a growing zoonotic disease in countries where consumption of raw/marinated fish is high. Moreover, Anisakiasis could be under diagnosed in countries where the consumption of these dishes is less common, since it could be easily misdiagnosed as appendicitis, gastric ulcer or other food allergies. Allergen structural studies are essential for the development of specific diagnostic tests and novel immunotherapy strategies. In the present study, we have elucidated for the first time the tertiary structure of Ani s 5 *Anisakis* allergen and its IgE and IgG_4_ regions implicated in allergic response. Ani s 5 belongs to the SXP/RAL-2 protein family. Several members of this family have been detected in animal and plant parasitic nematodes. As no homologs have been identified outside the Nematoda, these proteins may be suitable targets for controlling the damage caused by these parasites. Our work reveals that the structure of Ani s 5 resembles that of Calmodulin but binds Mg^2+^ instead of Ca^2+^, which suggests some putative functions for SXP/RAL-2 proteins.

## Introduction

Consumption of lightly cooked seafood is growing in developed countries since it is regarded as healthy food. This, together with the high rates of parasitation of fish worldwide [1], makes infections by the parasitic nematode *Anisakis* a serious health hazard. In fact, the number of cases of Anisakiasis is increasing in countries like Spain, Italy and Japan where consumption of raw or lightly cooked fish is high [2], [3], [4]. However, the frequency of the disease could be underestimated in other countries where the consumption of these dishes is less frequent as it can be easily misdiagnosed as appendicitis, gastric ulcer or other food allergies [4].

Anisakiasis is caused by nematodes of the genus *Anisakis*, mainly *A. simplex* and *A. pegreffii*. The disease is the result of the accidental ingestion of third stage larvae of the parasite found in raw or undercooked marine fish [4]. Clinical symptoms include severe epigastric pain, nausea and vomiting, occurring a few hours after the ingestion of fish, accompanied by severe allergic reactions such as angioedema, urticaria or anaphylaxis [5], [6]

Thirteen *Anisakis* proteins have been described as being responsible for the severe IgE mediated allergic reactions (www.allergen.org), and some of them are used for component resolved diagnosis *in vitro* to improve the specificity of the tests [7] Approximately 25–40% of *Anisakis* allergic patients, particularly those who suffer allergic symptoms following ingestion of well-cooked or canned fish have IgE against the Ani s 5 antigen [8]; furthermore, the specificity of diagnostic tests designed to detect antibodies against Ani s5 is greater than 95% [7].

Designing improved diagnostic tests and specific immunotherapy without unwanted side effects requires the elucidation of the interaction between allergens and the immune system at the molecular level.

Knowledge of the 3D structure of the allergens as well as the identity of their IgE binding epitopes (since IgE are responsible for the allergic symptoms) is pivotal to achieve this goal and, in turn, contribute to a better understanding of the relationships between protein structure and allergenicity. Similarly, the identification of IgG_4_ epitopes is becoming crucial, since this immunoglobulin has been associated with suppression of IgE-dependent immediate hypersensitivity reactions which occur, for example during helminth infection [9].

In addition, the determination of a protein's structure can also provide a basis to better predict or understand its biological function. The function of Ani s 5 in *Anisakis* larvae is unknown. It is a member of the SXP/RAL-2 protein family which includes proteins from various nematode species, such as AS16 protein from *Ascaris suum*, the antigens WB14 and SXP from *Wuchereria bancrofti*, and the immunodominant hypodermal antigen from *Onchocerca volvulus* [10]. The proteins belonging to this family contain the DUF148 domain (Pfam accession number PF02520) that comprises two conserved motifs: SXP1 and SXP2 [11]. This domain's function is unknown. Several members of this protein family have been described in animal parasitic nematodes [11], [12], [13], plant parasitic nematodes [14], [15] and have been predicted in the *C. elegans* genome (*i.e.* accession numbers P36991, Z82260, U42841, U39849, Z49073).

Since homologs of these proteins have not been identified outside the Nematoda, they may be suitable targets for the development of novel control strategies against the diseases caused by these parasites. Indeed, SXP/RAL2 proteins have been studied as potential microfilarial vaccines and exploited as antigens for the development of serological diagnostic assays [11].

Here, we have expressed Ani s 5 in *E. coli*, solved the high-resolution 3D structure in solution by NMR (Nuclear Magnetic Resonance) methods, deduced from it a functional feature, namely Mg^2+^ binding, and mapped its putative IgE and IgG_4_ epitopes using synthetic peptides. Finally, we have identified the position of these epitopes in the allergen's 3D structure.

## Materials and Methods

### Ethics statement

Approval from the Carlos III Hospital's Ethical Committee and written informed consent from the patients were obtained.

### Patients and sera

Sera from nine patients allergic to *Anisakis* selected at the Allergy unit of the Hospital Carlos III (Madrid, Spain) were included. Patients were selected on the basis of the following criteria: (i) clinical history of allergy to *Anisakis*; (ii) positive skin-prick test response to *Anisakis* extract and ImmunoCAP classes 3–6 to *Anisakis*; and (iii) positive reaction to Ani s 5 by IgE-immunoblotting. A pool of sera from 5 atopic patients allergic to multiple pollens was used as negative control.

### Cloning, heterologous expression and purification of recombinant Ani s 5 in *E. coli*

Total RNA from *Anisakis* larvae was extracted with Trizol (Gibco, Invitrogen, Carlsbad, CA, USA). The cDNA was synthesized using the Omniscript RT kit (Qiagen, Venlo, Netherland). The coding region of Ani s 5, except the signal peptide, was amplified by PCR using two primers based on the known cDNA sequence of Ani s 5 (accession number AB274998), a sense primer 5′-GACGACGACAAGATGGACGATACTCCCCCT-3′ and antisense primer 5′-GAGGAGAAGCCCGGTTATTGAGGCACAG-3′. These primers included the appropriate sequence (underlined) for directional cloning into the plasmid expression vector pET46 EK/LIC (Novagen, Merck KGaA, Darmstadt, Germany). This plasmid produces a protein with a histidine N-terminal tag for affinity purification. The resultant plasmid was transferred into *E. coli* BL21 Star (DE3) One Shot (Invitrogen, Carlsbad, CA, USA).

The general protocol for the production of isotopically labelled protein is as follows. Transformed bacteria were grown in 1 L of LB medium supplemented with ampicillin 50 µg/ml at 37°C shaken at 300 rpm. Upon reaching optical density at 600 nm of 0.7, the cells were pelleted by a 30 min centrifugation at 5000× g and washed with M9 salt solution (KH_2_PO_4_ 5 g/l, Na_2_HPO_4_-7H_2_O 16 g/l, NaCl 0.5 g/l). The cell pellet was resuspended in 250 ml of isotopically labelled minimal medium (M9 salt solution plus 2 mM MgSO_4_, 0.1 mM CaCl_2_, 2.5 mg/l Biotin, 0.5 mg/l Thiamine, 50 µl/l LB), and 4 g ^13^C-D-glucose and 1 g ^15^NH_4_Cl per litre (Tracer, Madrid, Spain) as unique sources of carbon and nitrogen respectively [16]. After 1 h at 37°C to allow the recovery of growth and clearance of unlabeled metabolites, protein expression was induced by addition of 0.8 mM IPTG. The cells were harvested after 4 h.

*E. coli* cells were pelleted and resuspended in binding buffer (50 mM Tris; 400 mM NaCl; 20 mM imidazole; 1 mM β-mercaptoethanol) plus lysozyme and protease inhibitors. The suspension was sonicated on ice to obtain the lysate. Insoluble material was removed by centrifugation at 16,000×g for 10 min at room temperature. Recombinant isotope labelled Ani s 5, expressed as a 6-His tagged protein, was purified by a two-step procedure carried out on a liquid chromatography system ÄKTAdesign™ (GE Healthcare Bio-Sciences AB. Uppsala, Sweden). First, it was purified from the soluble protein fraction using HisTrap HPcolumn (GE Healthcare Bio-Sciences AB), following manufacturer's specifications. The fractions containing the allergen were pooled and dialyzed against 20 mM Tris pH 8. Subsequently, rAni s 5 was purified by anion-exchange chromatography on a HiTrap Q HP column (GE Healthcare Bio-Sciences) equilibrated with 10 mM Tris buffer pH 8 and elution was performed with a linear gradient of NaCl. Protein concentration was determined by the Bradford method (Bio-Rad, Hercules CA, USA).

For biophysical determinations, unlabeled protein was used and the His-tag was cleaved by incubation with enterokinase (NEB, Ipswich MA, USA) overnight at 23°C and the tail was eliminated by exhaustive filtration in an Amicon Ultra 0.5 Ultracel (10 kDa cutoff) (Millipore, Billerica MA, USA).

### NMR samples, assignment and dynamics

Typically, NMR samples contained approximately 0.5 mM of labelled protein (containing the histidine N-terminal tag) prepared in both 90% H_2_O/10% D_2_O and D_2_O at pH 5.0 and 3.5 (uncorrected for deuterium isotope effects). Sodium-4,4-dimethyl-4-silapentane-1-sulfonate (DSS) was used as internal ^1^H chemical shift reference. Other conditions were tested: higher protein concentrations and higher pH values rendered broader signals and NMR spectra of lower quality for structural purposes. All spectra were recorded on a Bruker AV-800 spectrometer equipped with a cryoprobe at 25° and 35°C. The assignment of ^1^H, ^13^C and ^15^N resonances was achieved using a standard suite of heteronuclear 2D and triple resonance 3D spectra: ^1^H-^15^N-HSQC, ^1^H-^13^C-HSQC, HN(CO)CA, HNCA, CBCA(CO)NH, CBCANH, HNCO, HC(C)H-TOCSY, (H)CCH-TOCSY, HACANH, HBHACONH, ^1^H-^13^C-NOESY (aliphatic and aromatic regions) and ^1^H-^15^N-NOESY as previously described [17]. The ^1^H, ^13^C and ^15^N chemical shifts have been deposited in the BioMagResBank (http://www.bmrb.wisc.edu/) under the accession number 19372. Conventional ^1^H-^15^N heteronuclear relaxation NOE (Nuclear Overhauser Effect) data were also determined. The experiments with and without proton saturation were acquired simultaneously in an interleaved manner with a recycling delay of 7 s, and were split during processing into separate spectra for analysis. The values for the heteronuclear NOEs were obtained from the intensity ratio of the resonances according to: I_sat_/I_ref_. The uncertainty was estimated to be about 5%. Spectra were processed with TOPSPIN (Bruker Biospin, Karlsruhe, Germany) and analyzed with SPARKY [18]. The program TALOS+ [19] was used to predict the backbone order parameter S^2^ from the random coil chemical shifts and the experimental values obtained in the assignment process of Ani s 5.

### Structure calculation

The solution structure of Ani s 5 was calculated with the program CYANA 2.1 [20] based on experimental NOE-derived distance constraints and TALOS+-derived dihedral constraints [19]. The calculation statistics are summarized in Table 1. We used a combination of automatic runs based on the standard 7-cycle iterative process and non-automatic/manual runs to solve NOE ambiguities derived from the large signal overlap. This protocol was useful to largely increase the set of assigned non-sequential NOEs that defined the secondary structure and the protein fold. Finally, 100 structures were generated in the last standard, non-automatic run on the basis of 1454 upper distance constraints obtained from a combination of lists of manually assigned and automatically assigned NOEs from previous runs. Hydrogen bonds detected from deuterium exchange-protected HNs were introduced in the helices. The utility of these experimentally derived constraints to better define long helices such as H3 has been has been previously demonstrated for the case of apo Calmodulin [21]. The 20 conformers with the lowest target function values were selected for further refinement and were finally minimized with AMBER [22] using the Gibbs-Boltzmann continuum solvation model [23]. The structure has been deposited in the Protein Data Bank under the accession code 2mar. MOLMOL [24] was used for molecular display.

**Table 1:** - Structural statistics of the 20 best NMR structures of Ani s 5.

**NOE Distance and Dihedral Constraints**	
N°. of intra residual distances	446
N°. of sequential distances	469
N°. of medium-range distances (1<|i−j|<5)	475
N°. of long-range distances (|i−j|≥5)	64
N°. of angular restraints (φ, ψ)	208
N°. of total restraints	1662
**Structure Calculation**	
Average CYANA target function value	8.0 (7.13 to 9.29)
Average maximum distance violation (Å)	0.35 (0.23 to 0.87)
Average maximum dihedral angle violation (°)	4.23 (3.39 to 5.30)
AMBER total energy (kcal/mol)	−4447 (−4523 to −4394)
van der Waals (kcal/mol)	−924 (−955 to −889)
Electrostatic (kcal/mol)	−10595 (−11276 to −10193)
**RMSD (Å)**	
Bond lengths from ideal geometry	0.0096±0,0002
Bond angles from ideal geometry	2.39±0.04
Pairwise backbone (1–134)	7.5±2.5
Pairwise backbone (15–113)	2.2±0.6
Pairwise backbone (15–64))	1.0±0.3
Pairwise backbone (65–113)	1.6±0.7
**Ramachandran Plot Analysis (%)**	
Most favored regions	87.5
Additional allowed regions	11.7
Generously allowed regions	0.7
Disallowed regions	0.2

### Biophysical characterization

For all biophysical experiments, the samples were prepared in water with or without CaCl_2_ or MgCl_2_ at different concentrations (from 10 to 300 mM). The pH was adjusted to 7, and the measurements were performed at 25°C. Circular dichroism experiments were carried out on a J-810 dichroism spectrometer (Jasco, Tokyo, Japan). The ANS (8-anilino-1-naphthalenesulfonic acid) fluorescence experiments were recorded in a Fluoromax-4 spectrofluorometer (Horiba Jobin Yvon, Kyoto, Japan). ANS was used at a final concentration of 100 µM. The concentration of Ani s 5 was 0.2 µM or 10 to 50 µM in fluorescence and in circular dichroism experiments, respectively. Sedimentation velocity experiments were performed on a Beckman-Coulter OPTIMA XL-1 (interference optics) analytical ultracentrifuge at 20°C. Measurements were performed under the NMR conditions and a velocity of 45,000 rpm. Differential sedimentation coefficients, c(s), were calculated by least squares boundary modeling of sedimentation velocity data using the program SEDFIT [25]. The SEDNTERP program [26] was used to calculate protein specific volumes from the amino acid sequences, and the buffer viscosity and density.

### SDS-PAGE

15% SDS-PAGE gels were prepared and run using the conventional protocol but in the absence or presence of 2 mM EDTA, 2 mM CaCl_2_ or 2 mM MgCl_2_ in all solutions: stacking gel, separation gel, loading buffer and running buffer and markers (Precision plus protein standards, Bio-Rad, Hercules CA, USA).

### Peptide microarray-based immunoassay

A library of 20-mer peptides scanning the whole primary sequence of Ani s 5 with a 3 residue offset was commercially synthesized and printed in triplicate onto epoxy-derivatized glass slides. Mouse and human IgG, as well as anti-human IgE and IgG_4_ antibodies, were co-immobilized to serve as assay controls (JPT Technologies GmbH, Berlin, Germany).

The slides were rinsed with TBS-0.1% Tween20, and nonspecific binding sites were blocked with 1% SmartBlock (CANDOR Bioscience GMBH, Wangen, Germany) in TBS-T for 1 hour. After removing the blocking solution, the slides were incubated for 2 hours with patient sera diluted 1∶10 in SuperBlock T20 (Thermo Scientific/Pierce, Rockford, IL USA) with agitation. Subsequently, the slides were incubated for 1 hour with monoclonal mouse antihuman IgE (Ingenasa, Madrid, Spain) or antihuman IgG_4_ (Sigma) diluted to 1 µg/ml. DyLight649 labelled anti-mouse IgG (Thermo Scientific/Pierce) (1 µg/ml) for 45 min was used as detection antibody. All steps were performed on a TECAN HS4X00 microarray processing station.

Finally, slides were washed thoroughly with TBS-0.1% Tween20 and SSC buffer-0.05% Tween20, dried using a steam of nitrogen gas and examined with a high resolution microarray scanning system (Axon Genepix Scanner 4200AL). Images were saved as TIF files.

Regarding the data analysis, the mean and median intensities and background intensity for each spot-feature were determined with GenepixPro 7.0 Spot-recognition software package. All displayed diagrams were corrected for local background of each feature, according to the algorithm applied in the spot recognition software. The data evaluation software calculates the signal intensities for pixels within recognized spots (feature) and signal intensities for pixels around recognized spots (background). The mean background intensity was subtracted from the median of feature intensity resulting in corrected median values (signal minus background). The mean values of the corrected median of signal intensities from three identical microarray images were used for data evaluation. Mean values of signal intensities were expressed in Light Units (LU) and exported to Microsoft Excel. The reactivity of each individual serum was plotted after subtracting the negative pool of sera reactivity to each peptide. To establish a cut-off, the Light Units values were ranked, the lowest 25% of the values were averaged and 3 times the standard deviation of the lowest 25% was added to this average [27].

### Dot-blot analysis of solid phase-bound synthetic peptides (SPOTs)

Forty-two synthetic dodecapeptides overlapping by 9 amino acids spanning the complete Ani s 5 protein sequence and covalently bound to a cellulose membrane (SPOTs) were commercially obtained from the Spanish National Centre of Biotechnology (Madrid, Spain). The SPOTs membranes were blocked with buffer containing 5% BSA for three hours at room temperature and then incubated overnight with sera from *Anisakis* allergic patients (1∶5 dilution). After washing the membrane with TBS-0.1% Tween20, IgE-binding was detected with horseradish peroxidase-labelled mouse antihuman IgE monoclonal antibody (SouthernBiotech, Birmingham, AL, USA) 1∶5000 diluted in TBS-0.5% Tween20. The peroxidase reaction was developed with ECL western blotting reagent according to the manufacturer's instructions (Amersham Biosciences, Little Chalfont, UK).

### Accession numbers

Ani s 5 sequence:

http://www.ncbi.nlm.nih.gov/genbank/; accession number: AB274998

Ani s 5 structure:

http:/www.bmrb.wisc.edu; accession number: 19375

## Results

### NMR assignments

Prior to the structural and biophysical characterization, we demonstrated that Ani s 5 is a monomer in the NMR conditions. Sedimentation velocity experiments yielded results compatible with a single monomer species. The average molecular mass of Ani s 5 resulting from the centrifugation analysis was 16.8 kDa, in close agreement with the theoretical mass value (17.5 kDa) calculated from its amino acid sequence (with His-tag) and 100% ^13^C and ^15^N isotopic labelling (supplementary Fig. S1). NMR backbone assignment was performed following conventional strategies and nearly all Cα and HN resonances were identified. The HN signals of A67 and A106 could not be observed in our experimental conditions due to broadening effects probably related with the specific dynamic properties of this protein. Also, the assignments of three consecutive prolines: P5, P6 and P7, are missing. Most of side chain ^1^H, ^13^C and ^15^N chemical shifts were also assigned.

### NMR structure of Ani s 5

**Figure 1:**
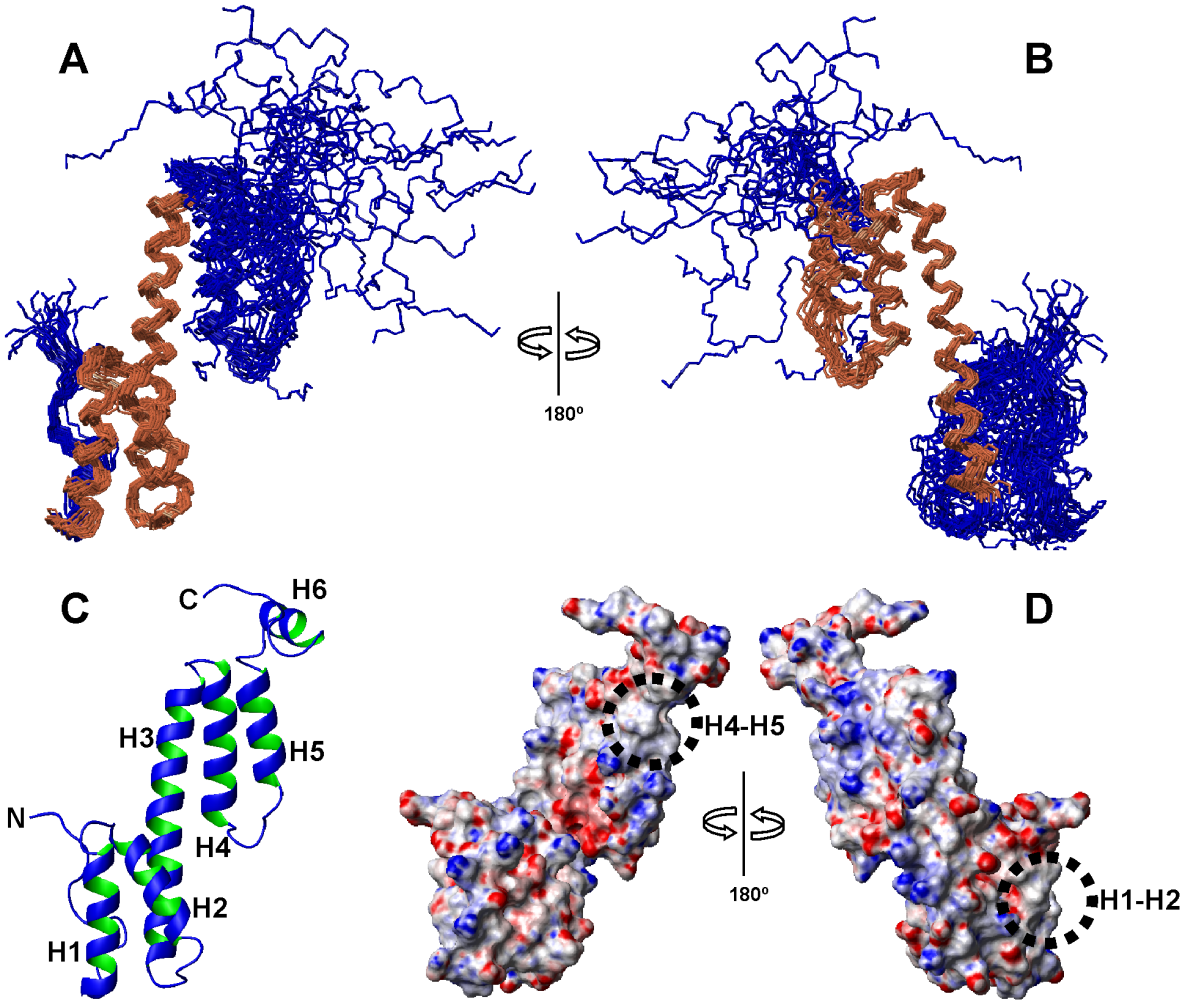
Solution structure of Ani s 5. **A**, Backbone superposition of helices H1–H3 of the 20 lowest-energy conformers of Ani s 5 obtained by NMR. The superimposed helices are in gold and the rest of the protein is in blue. **B**, Backbone superposition of helices H3–H5 of the 20 lowest-energy conformers of Ani s 5 obtained by NMR. The superimposed helices are in gold and the rest of the protein in blue. The orientation of the ensemble is rotated 180° in **A** and **B**. **C**, Ribbon display of a representative conformer of the family showing the positions of the helical segments (H1 to H5) and one of the possible orientations of the C-terminal tail containing helix H6, with respect to the protein core. Helices H1 (15–27), H2 (33–47), H3 (51–77), H4 (82–96), H5 (103–113), H6 (118–125) and the N- and C-termini are labeled. **D**, Electrostatic surface of Ani s 5. Negative charges are in red, positive charges are in blue. Circles indicate the hydrophobic regions at the level of H1–H2 and H4–H5 helices. Left, the protein has the same orientation as in A. Right, 180° rotated view.

The determination of the solution structure of Ani s 5 was challenging due to a combination of limited spectral dispersion (common to all-helical structures), the presence of broad signals and large signal overlap (supplementary Fig. S2). Hence, using a strategy which combined runs of automated CYANA calculations that allowed additional NOEs to be assigned, with standard CYANA calculations that included lists of automatically assigned NOEs together with manually derived distances from unambiguously assigned NOEs, we succeeded in determining the structure of Ani s 5 to high-resolution. The structure (Fig. 1) reveals an all-helical protein consisting of a disordered N-terminal tail (residues 1–14), a long central helix (helix 3, H3 residues 51–77) and two groups of two shorter helices at its N-terminus (helix 1, H1 residues 15–27; helix 2, H2 residues 33–47) and towards its C-terminus (helix 4, H4 residues 82–96; helix 5, H5 residues 103–113). Finally, there is a short, disordered helix (helix 6, H6 residues 118–125) right at the C-terminus. This disorder is probably related to the lack of assigned long-range restraints (|i−j|>5 residues) with the rest of the structure due to signal overlap and chemical shift degeneration. The helices are all distributed longitudinally with small tilted angles and an approximate 90° rotation between the N-terminal and C-terminal portions. Their relative orientation was determined using the CYANA protocol described in Materials and Methods, which led to a significant set of long-range restraints (|i−j|>5) compatible with the structure solutions. The position of helices H1 and H2 is determined by the contacts between the side chains of V17 and L47; A20 and W43; F21 and L40; L24, L40 and W43; and L25 and L40; that of helices H2 and H3 by the side chain and backbone contacts between P34 and M62; and V44, K52 and F55; that of helices H3 and H4 between A67, E96 and A95; A70 and S92; and V77 and K85; finally that of helices H4 and H5 by the contacts between E90 and I103. All the Pro residues are in the *trans*- configuration. The backbone RMSD (Root Mean Square Deviation) excluding the disordered N-terminal and helix H6 is 2.18 Å (residues 15–113). The local structure is better defined as shown by smaller RMSD values for the N- and C-terminal half portions of 0.99 Å (residues 15–64) and 1.55 Å (residues 65–113). Almost all the backbone Φ and ψ angles (99.2%) lie in the most favored regions of the Ramachandran plot.

Figure 1D shows the surface representations of the electrostatic potential of Ani s 5. In addition to the positive and negative charges distributed along the protein surface; two patches lacking charges can be seen. These patches correspond to the H1–H2 and H4–H5 helix interfaces. Interestingly, the loops connecting these helix pairs contain residues (D28, D29, E33 in loop connecting H1 and H2; and D96, D97, N100 in loop connecting H4 and H5) that are abundant in different cation binding motifs.

Nearly all residues are exposed to the solvent with ASA (Accessible Surface Area) >20%. Those buried in the structure (ASA<5%) are mainly hydrophobic, in particular F21, I36, E37, L40, D41, V44, A70 and A95. Hydrophobic contacts between helices play an important role in sustaining the structure of Ani s 5, as commented before. Also, hydrophobic surfaces could be relevant for its function as we will propose later on. There are a few charge-charge interactions involving the H73-D88 and E74-K85 pairs in helices H3 and H4, and the side chains of D33 and K65 in helices H2 and H3 are close enough to interact.

### NMR dynamics

**Figure 2:**
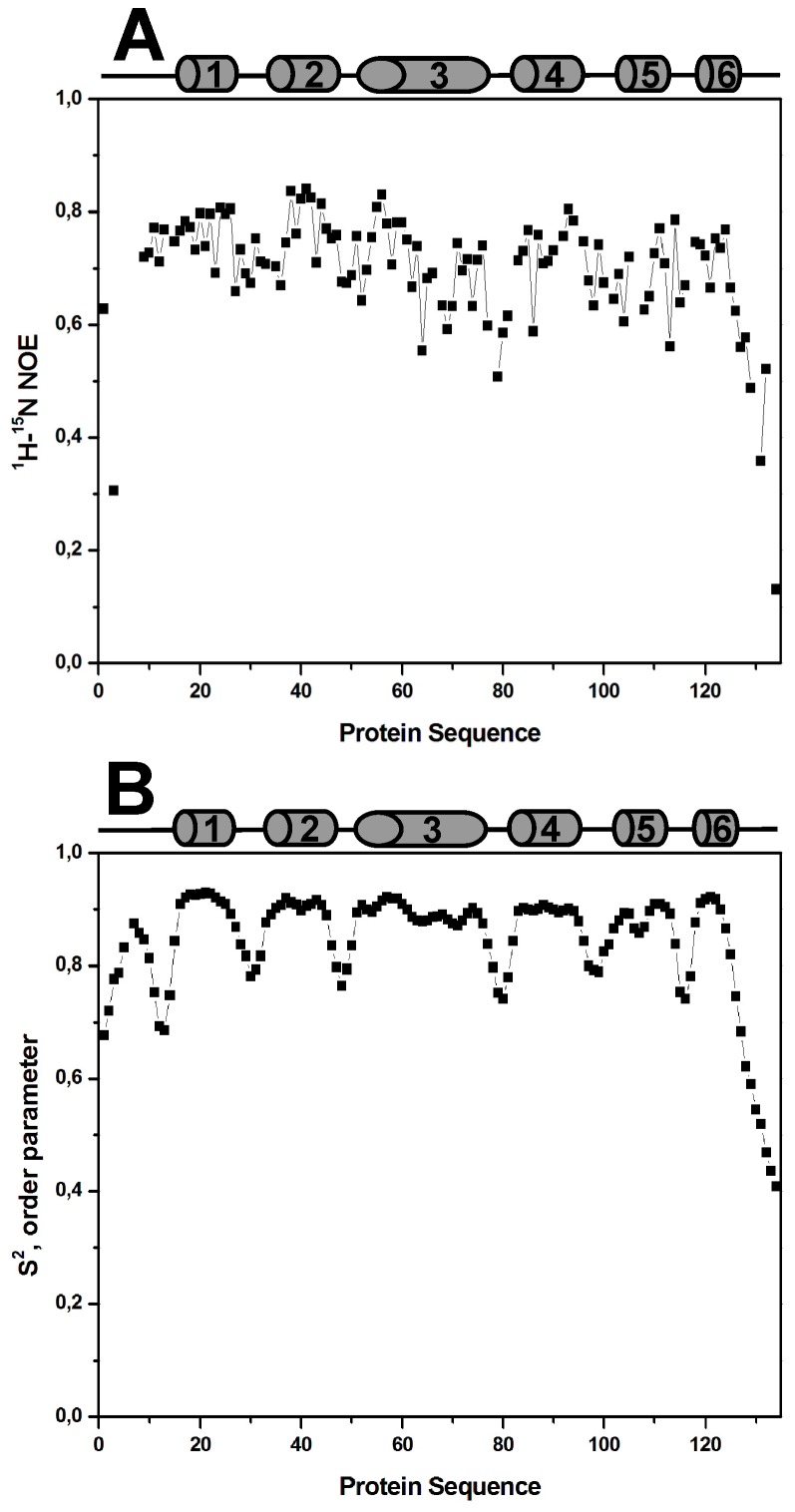
Dynamic data of Ani s 5. **A**, Heteronuclear NOE values as a function of the protein sequence. Black squares represent the experimental values. **B**, S^2^ order parameters obtained from NMR chemical shifts by the program TALOS+ [19].

We have measured the heteronuclear NOEs to get information on the local backbone flexibility of Ani s 5 in the ns-ps time-scale (Fig. 2A). The protein is, in general, highly dynamic with a mean NOE value of 0.7. The data corroborate the disordered nature of the N- and C-terminal regions, and show that their high flexibility could be responsible for the absence of long-range NOEs.

We have also applied an algorithm based on chemical shift data that yields accurate, residue level data on the protein backbone mobility [28]. The results of the calculated backbone order parameters S^2^ are shown in Fig. 2B and match quite well those obtained from NOE data, indicating that H6's lack of orientation is not due to high flexibility of the segment linking H6 to H5. Unfortunately, the large signal overlap present in the ^1^H-^15^N and ^1^H-^13^C HSQC spectra, prevented the measurement of a sufficient number of RDCs (Residual Dipolar Coupling) for residues in helix H6, and thwarted our attempts to better define its orientation with respect to the rest of the protein.

### Divalent cation binding

The fold of Ani s 5 is reminiscent of the structure of Calmodulin, a calcium binding protein. Since no knowledge exists so far about the function of Ani s 5 or any member of the DUF148 domain family, we decided to explore the possible significance of this structural similarity.

**Figure 3:**
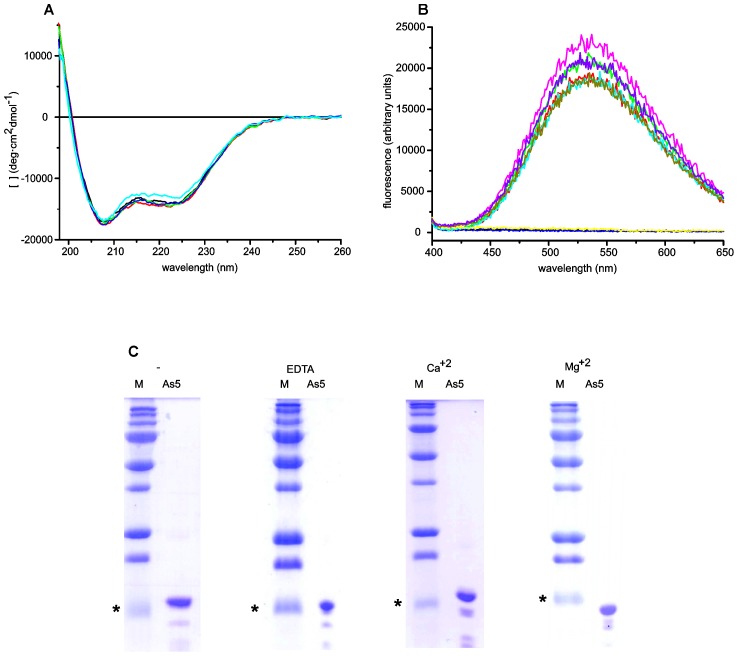
Biophysical characterization of Ani s 5 and effect of divalent cations. **A**, Far-UV CD spectra of Ani s 5 in the absence (black) or in the presence of different cation concentrations: red, MgCl_2_ 2 mM; green, MgCl_2_ 20 mM; blue, MgCl_2_ 100 mM; cyan, CaCl_2_ 100 mM at pH 7. **B**, Fluorescence emission spectra after excitation at 350 nm of Ani s 5 (black), ANS (red), Ani s 5 with CaCl_2_ (dark blue), Ani s 5 with MgCl_2_ (yellow), ANS with Ani s 5 (green), ANS with CaCl_2_ (cyan), ANS with MgCl_2_ (olive green), Ani s 5 with ANS and CaCl_2_ (magenta), and Ani s 5 with ANS and MgCl_2_ (violet). **C**, SDS-PAGE of Ani s 5, Ani s 5 with EDTA, 2 mM CaCl_2_ or 2 mM MgCl_2_ 2. M denotes the molecular weight markers used and the asterisks indicate the 15 kDa standard.

As a first approach, we studied Ani s 5 by circular dichroism. The data from the far-UV CD (circular dichroism) spectra indicate that the protein has a high helical content and no changes in the secondary structure were induced by the addition of Ca^2+^ or Mg^2+^ (Fig. 3A).

Also, we carried out fluorescence experiments in the presence of ANS which is a specific probe for hydrophobic patches in molten globule and non-compact states [29]. As shown in Fig. 3C, only a similar slight increase of the signal (around a 10%) is detected in all the conditions suggesting that Ani s 5 is a well-packed, well folded protein.

Finally, we studied the mobility of Ani s 5 in SDS-PAGE gels in the presence or absence of chelating agents, Ca^2+^ or Mg^2+^ (Fig. 3D). It has been reported that divalent cation-binding proteins shift and run faster in gels containing those ligands [30]. The relative mobility of the markers adjusted to a second order exponential decay curve for all gels (data not shown), and the interpolation of Ani s 5's mobility showed an apparent molecular mass of 15.7–15.8 kDa (theoretical molecular weight of the unlabeled Ani s 5: 14.9 kDa) for all the conditions except in the presence of MgCl_2_. In this case, the band runs faster and yields an apparent molecular weight of 14 kDa. This clear increase of Ani s 5 mobility, strongly suggests the capability of Ani s 5 to specifically bind Mg^2+^.

### IgE binding epitope characterization

**Figure 4:**
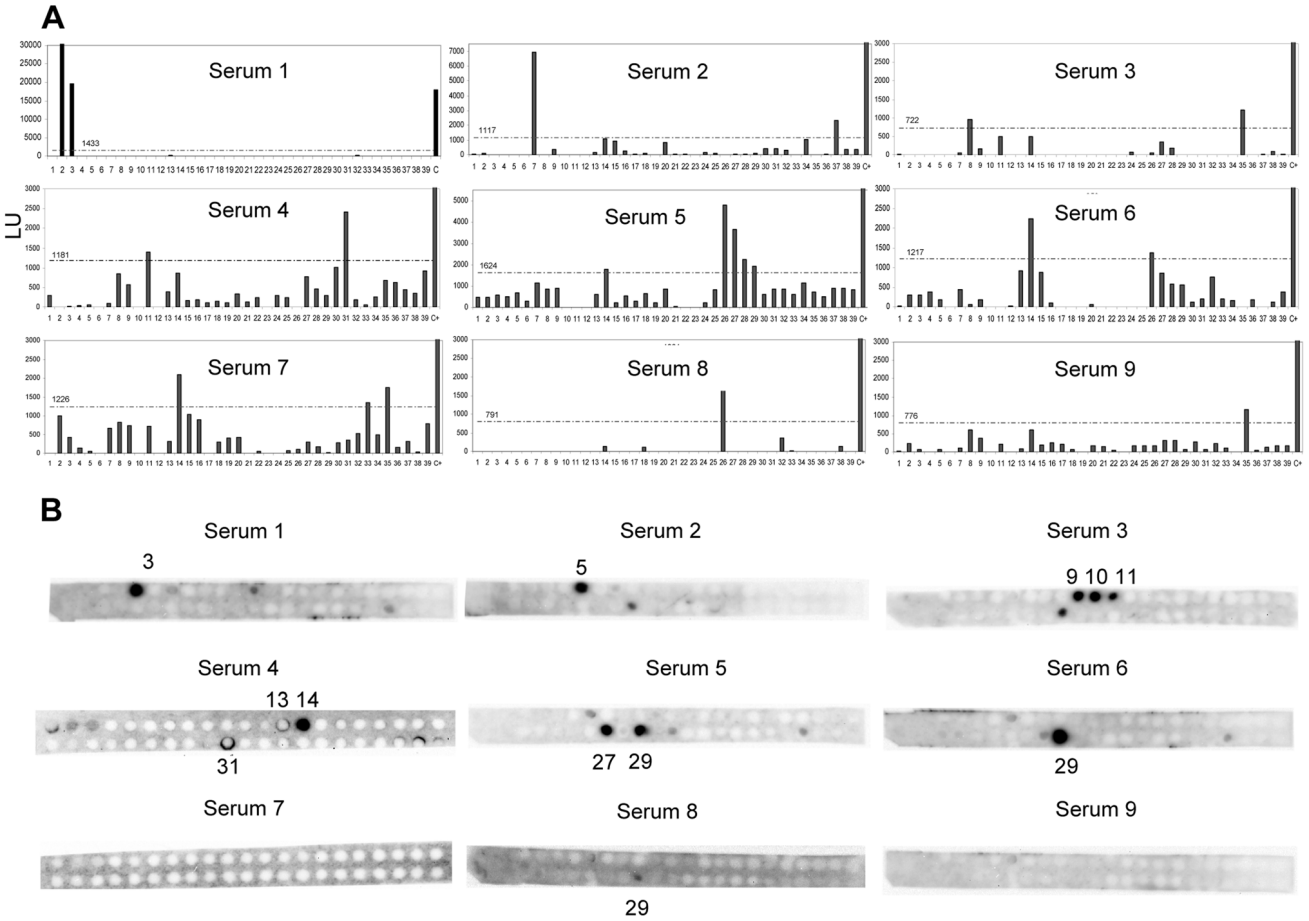
IgE binding epitope characterization. **A**, Average signal intensity (in light units) of the peptide-based microarray immunoassay for the nine Ani s 5 positive sera tested, cut-off values are indicated above the dotted line. **B**, IgE immunodetection of SPOTs membranes for the same nine Ani s 5 positive sera.

First, a set of 20-mer overlapping synthetic peptides with an offset of 3, corresponding to the sequence of Ani s 5 was used in a microarray-based immunoassay. These peptide microarrays were assayed with sera from nine individual *Anisakis* allergic patients. The IgE binding intensity was highly variable among the sera tested; however it was possible to identify 11 IgE binding regions (Table 2 and Fig. 4A): region E1 covers positions P4-K26 (peptides 2, 3), E2 positions K19-K38 (peptide 7), E3 positions F22-D41 (peptide 8), E4 positions K31-D50 (peptide 11), E5 positions L40-K59 (peptide 14), E6 positions A76-A95 (peptide 26), E7 positions K79-Q104 (peptides 27 to 29), E8 positions L91-Q110 (peptide 31), E9 positions D97-L116 (peptide 33), E10 positions I103-D122 (peptide 35) and E11 positions I109-I128 (peptide 37).

**Table 2:** IgE binding epitopes detected. The minimum unit necessary for IgE recognition is shown in bold and the residues forming alpha helix are underlined.

Location of peptides	Epitope	Microarray peptide	SPOTs peptide	Amino acid sequence	Sera
	E1	2		4-PPP**PPFLAGAPQDVV** KAFFE-23	1
		3		7-**PPFLAGAPQDVV** KAFFELLK-26	
α helix			3	7-**PPFLAGAPQDVV** -18	
H1(15–27)	E2		5	13-APQDVV**KAFFEL**-24	2
		7		19-**KAFFEL** LKKDETKTDPEIEK-38	
	E3	8		22-FELLKKDET**KTDPEI** EKDLD-41	3
			9	25-LKKDET**KTDPEI**-36	
			10	28-DET**KTDPEI**EKD-39	
α helix			11	31-**KTDPEI** EKDLDA-42	
H2 (33–47)	E4	11		31-KTDPEIEKD**LDAWVDTL** **G**GD-50	4
			13	37-EKD**LDAWVDTL** **G**-48	
			14	40-**LDAWVDTLG**GDY-51	
α helix	E5	14		40-LDAWVDTLGGDYKAKFETFK-59	5, 6, 7
H3 (51–77)					
	E6	26		76-AVAKMTPEA**KKADAELSKIA**-95	5, 6, 8
			29	85-**KKADAELSKIA** E-96	
		26		76-AVAKMTPEA**KKADAE**LSKIA-95	5
α helix	E7	27		79-KMTPEA**KKADAE**LSKIAEDD-98	
H4 (82–96)		28		82-PEA**KKADAE**LSKIAEDDSLN-101	
		29		85-**KKADAE** LSKIAEDDSLNGIQ-104	
			27	79-KMTPEA**KKADAE**-90	
			29	85-**KKADAE** LSKIAE-96	
	E8	31		91-**LSKIAEDDSLNG**IQKAQKIQ-110	4
			31	91-**LSKIAEDDSLNG**-102	
α helix	E9	33		97-DDSLNGIQKAQKIQAIYKTL-116	7
H5 (103–113)					
α helix	E10	35		103-IQKAQKIQAIYKTLPQSVKD-122	3, 7, 9
H6 (118–125)	E11	37		109-IQAIYKTLPQSVKDELEKGI-128	2

A complementary approach was explored analyzing SPOTs membranes with 12-mer synthetic peptides (overlapping 9 amino acids), which spanned the entire allergen sequence (Table 2 and Fig. 4B). Seven IgE binding regions were detected testing the SPOT membranes with the same sera used in microarray experiments. The main responses were observed for peptide 3 (positions P7-V18), peptide 5 (positions A13-L24), peptides 9 to 11 (positions L25-A42), peptides 13 and 14 (positions E37-Y51), peptide 27 (positions K79-E90), peptide 29 (positions K85-E96) and peptide 31 (positions L91-G102).

The correlation of peptide microarray and SPOTs membrane experiments was good and highly consistent for each serum. However, there were some discrepancies as sera 7 and 9 rendered negative results with SPOTs membranes (Fig. 4B). Specifically, none of the microarray peptides 14, 33, 35 and 37 gave positive results by the SPOTs technique (Table 2).

Combining the results from both techniques, for some IgE epitopes it was possible to predict the minimal putative polypeptide segment required for IgE binding to Ani s 5 (Table 2, letters in bold).

Each serum recognized one or two epitopes. Three regions: E5 (microarray peptide 14), E6 (microarray peptide 26 and SPOT peptide 29) and E10 (microarray peptide 35) were recognized by 33.3% of the patients, and the remaining regions were recognized by one serum each (Table 2).

### IgG_4_ binding epitope characterization

Most of the sera (7 out of 9) scored positive for IgG_4_ in the peptide microarray immunoassay (Table 3). IgG_4_ binding closely matched the IgE binding positive peptides; the only exceptions were serum 2 that recognized peptide 34 for IgG_4_ and serum number 3 that recognized peptide 11 for IgG_4_.

**Table 3:** Comparison between IgE and IgG_4_ binding peptides detected by microarray immunoassay for each serum tested. Discrepancies between IgE and IgG4 binding peptides are in bold.

Sera	IgE Binding peptides	IgG_4_ Binding peptides	Epitope
1	2 (P4- E23), 3 (P7-K26)	2 (P4-E23), 3 (P7-K26)	E1
	7 (K19-K38)	7 (K19-K38)	E2
2	37 (I109-I128)	37 (I109-I128)	E11
		**34 (L100-S119)**	----
	8 (F22-D41)	8 (F22-D41)	E3
3	-----------	**11 (K31-D50)**	E4
	35 (I103-D122)	35 (I103-D122)	E10
4	**11 (K31-D50), 31 (L91-Q110)**	neg	-----
5	**14 (L40-K59), 26 (A76-A95), 27 (K79-D98), 28 (P82-N101), 29 (K85-Q104)**	neg	------
6	14 (L40-K59)	14 (L40-K59)	E5
	**26 (A76-A95)**	-------------	-----
7	14 (L40-K59)	14 (L40-K59)	E5
	**35 (I103-D122)**	-------------	E10
8	26 (A76-A95)	26 (A76-A95)	E7
9	35 (I103-D122)	35 (I103-D122)	E10

### Location of IgE and IgG_4_ binding epitopes on Ani s 5 structure

**Figure 5:**
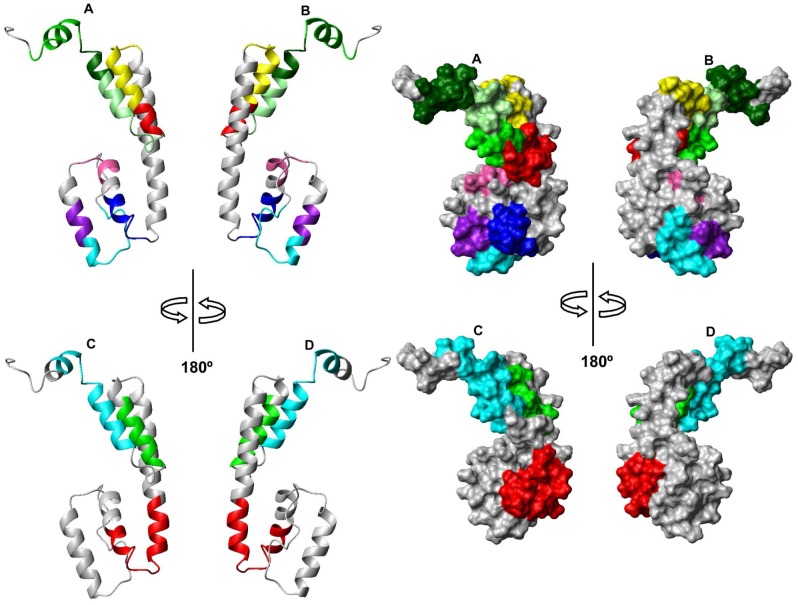
Ribbon and surface representations of Ani s 5 with epitope mapping. **A** and **B**, Peptide segments representing the minimum unit necessary for IgE/IgG_4_ recognition are colored in cyan (E1, 7–18), purple (E2, 19–24), hot pink (E3, 31–36), blue (E4, 40–48), yellow (E7, 79–90), red (E8, 91–102), green (E9, 97–116) and dark green (E11, 109–128). The overlapping region 110–116 is colored in pale green. **C** and **D**, Peptide segments recognized simultaneously by three different sera are colored in red (E5, 40–59), green (E6, 85–96) and cyan (E10, 103–122). Two perspectives rotated 180° with respect to each other are shown in each panel.

We have mapped the identified IgE and IgG_4_ epitopes on the protein surface (Table 2, 3 and Fig. 5). The first epitope E1 (P7-V18, recognized by serum 1) is located at the N-terminus, covering the first four residues of the alpha helix H1. The second epitope E2 (K19-L24) is located at H1. The third and fourth epitopes, E3 (K31-I36) and E4 (L40- G48), are located at the beginning and at the end of H2, respectively. There is a fifth epitope E5 (L40-K59) encompassing the last portion of H2, the first part of H3 and the corresponding connecting loop. We cannot define this epitope more precisely since sera did not react with any peptide in the SPOTs membranes. The epitopes E6 (K85-A95) and E7 (K79-E90) are both at the H4 helix in close proximity to each other. The boundary separating E6 and E7 is blurred as the minimum residues necessary for recognition are poorly resolved. However, we think there are two distinct epitopes since two different recognition patterns can be observed. The first pattern, corresponding to E6, is detected by sera 5, 6, and 8 that recognize microarray peptide 26 and SPOTs peptide 29. The second pattern is more complex as E7 is detected by serum 5 that recognizes microarray peptides 26, 27, 28, 29 and SPOTs peptides 27 and 29, but not peptide 28 (Fig 4B). The epitope E8 (L91-G102) is located just at the end of H4 and the loop that connects H4 and H5. The epitope E9 (D97-L116) spans H5 and some previous residues, E10 (I103-D122) is contained in H5, H6 and their connecting loop. Finally, epitope E11 (I109-I128) comprises a flexible portion of the protein, H6, and part of the C-terminus.

Overall, the structure of Ani s 5 is elongated with most of the amino acids accessible to the solvent. This likely account for why the epitopes identified here are distributed all over its structure, with the notable exception of the long central helix 3.

## Discussion

We report for the first time the three dimensional structure of an *Anisakis simplex* allergen and the location of its immunodominant regions.

We have determined the IgE and IgG_4_ linear epitopes using two complementary techniques. Firstly, we chose overlapping peptide microarrays of 20 amino acid length in order to improve the sensitivity of the microarray [31]. Secondly, we used overlapping peptide membranes (SPOTs) with 12-mer peptides to corroborate and more precisely define the epitopes. Both techniques have been profusely used to map many allergen epitopes [32]. The correlation between the two techniques was fairly good although with some discrepancies. We have described 11 IgE epitopes using microarrays and seven of them were also detected using the SPOTs technique. This discrepancy may be explained by the higher sensitivity of the microarray system [33]. Also, peptide lengths differed in both approaches, as well as the physico-chemical properties of the surface supports.

IgE plays a fundamental role in allergic diseases. Investigation of IgE-binding epitopes on allergen molecules is necessary to understand the allergen-antibody interaction [33]. This information helps in the development of more effective strategies for immunotherapy [34] and to improve *in vitro* allergy diagnosis [35]. In this sense, IgE binding to specific linear epitopes has been associated with the natural history of an individual's food allergy (i.e. transient *versus* persistent), and the diversity of epitope recognition has been associated with the severity of an allergic reaction [36], [37]. Likewise, it has been reported that some IgE-binding peptides from peanut allergens are “predictive peptides” that correlate with persistent symptomatic allergies, asymptomatic sensitization and outgrowing peanut allergy [38].

Regarding IgG_4_, the epitopes recognized were largely the same for each patient. A similar result has been reported for other peanut allergen, Ara h 2 [31]. This finding is consistent with the hypothesis that regulation of isotype switching is an important checkpoint in the development of clinical allergy *versus* tolerance and that IgG_4_ may act by blocking IgE access to the antigen [39].

It is well known that, thanks to the identification of immunologically relevant proteins by genomic projects and other approaches, the number of allergenic proteins has increased substantially, but in many cases their function remains unknown. This is the case of many allergenic proteins from nematodes. These immunological molecules act through the mucosal surfaces and share some characteristics such as resistance to heat and digestion [40], in fact Ani s 5 is heat-resistant [8]. In the case of allergy to *Anisakis*, the sensitization generally occurs when the parasite is still alive because heating or freezing the fish reduces the risk of allergy [41]. Therefore, we could expect that *Anisakis* allergens are involved in the invasion process (included in the ES products of the larva) or they are accessible to the immune system on the surface of the living parasite.

The 3D structure of Ani s 5 determined here is the first structure solved of an *Anisakis* allergen and also the first structure of an SXP/RAL-2 protein. In this context, this structure can be used for modelling the rest of the members of this protein family. Moreover, and more interestingly, we have been able to deduce and propose functional properties for Ani s 5, based on the structural similarity to other proteins and its capability to bind divalent cations. Given the complete lack of functional information about the SXP/RAL2 proteins, any findings are very valuable because they can lead to a more precise knowledge of the protein group and its importance in the life cycle of these parasites as well as offer clues as to why they are absent in other organisms.

Calmodulin binds Ca^2+^ through a helix-loop-helix structure with highly hydrophobic helices and negative residues arranged in a consensus manner in the loop [42]. Interestingly, we have located two hydrophobic patches in the surface of the Ani s 5 structure (Fig. 1D) corresponding to the H1–H2 and H4–H5 interfaces. We advance that the negatively charged side chains of some of the residues present in the loops connecting these helices could be involved in cation binding.

Hence, it seems that the pattern of an EF-hand-like functional motif, present in Calmodulin, is maintained in Ani s 5, even though the length of the loops are shorter and the exact positions of the acidic residues does not correspond to those present in the canonical EF-hand motif. This fact can account for the different specificity of Ani s 5 for Mg^2+^ in contrast to calmodulin related proteins which bind Ca^2+^.

It is worth pointing out that Ani s 8 [43] and Ani s 9 [44], other members of the SXP/RAL2 family, are also allergenic. This suggests that the function of these proteins can be important during the infection and the consequent allergic response.

As16, the homolog of Ani s 5 from *Ascaris suum and A. lumbricoides*, has been detected in all developmental stages of the parasite, including embrionated eggs, and among the ES (excretion/secretion products) products. Likewise, As16 was localized in the adult at the hypodermis, cuticle and the luminal surface of the intestine [45]. Ani s 5 has been also detected among the ES products released by the infective third-stage larvae and immunohistochemically located in the excretory gland, ventriculus and luminal surface of the intestinal epithelium [8]. Magnesium ions play key roles in stabilizing protein structures and membranes and are involved in the activation/inhibition of many enzymes [46]. The ubiquitous expression of As 16 and Ani s 5, lead us to propose that these proteins might act as transporters to different cellular locations where a high magnesium concentration can be required.

In conclusion, we have obtained the first structure of an allergen from *Anisakis simplex*, mapped its IgE and IgG_4_ epitopes and pinpointed their location on the protein surface. These results will help to elucidate the basis of this protein's allergenicity. Besides the evident immunological data that can lead to new therapeutic and diagnostics advances in allergy, the novel data we provide here open new paths to explore the possible roles of Ani s 5 and its family of proteins. Finally, the capability of Ani s 5 to bind Mg^2+^ demonstrated in this work importantly suggests a possible function for the SXP/RAL-2 group of proteins.

## Supporting Information

Figure S1 Characterization of Ani s 5 by analytical ultracentrifugation. Distribution of sedimentation coefficients measured at the NMR conditions.
(TIF)

Figure S2 ^1^H-^15^N HSQC spectrum of Ani s 5 recorded in a 800 MHz Bruker spectrometer at pH 3.5 and 35°C. Extensive signal overlap can be observed in the region ^1^H: 8.7–8.0 ppm, ^15^N: 122–117 ppm.
(TIF)
