# Supplementary figures and images for: Relationships between IgE/IgG4 Epitopes, Structure and Function in Anisakis simplex Ani s 5, a Member of the SXP/RAL-2 Protein Family
Source: PLoS Negl Trop Dis. 2014 Mar 6;8(3):e2735. doi: 10.1371/journal.pntd.0002735 (PMC3945735; doi:10.1371/journal.pntd.0002735)

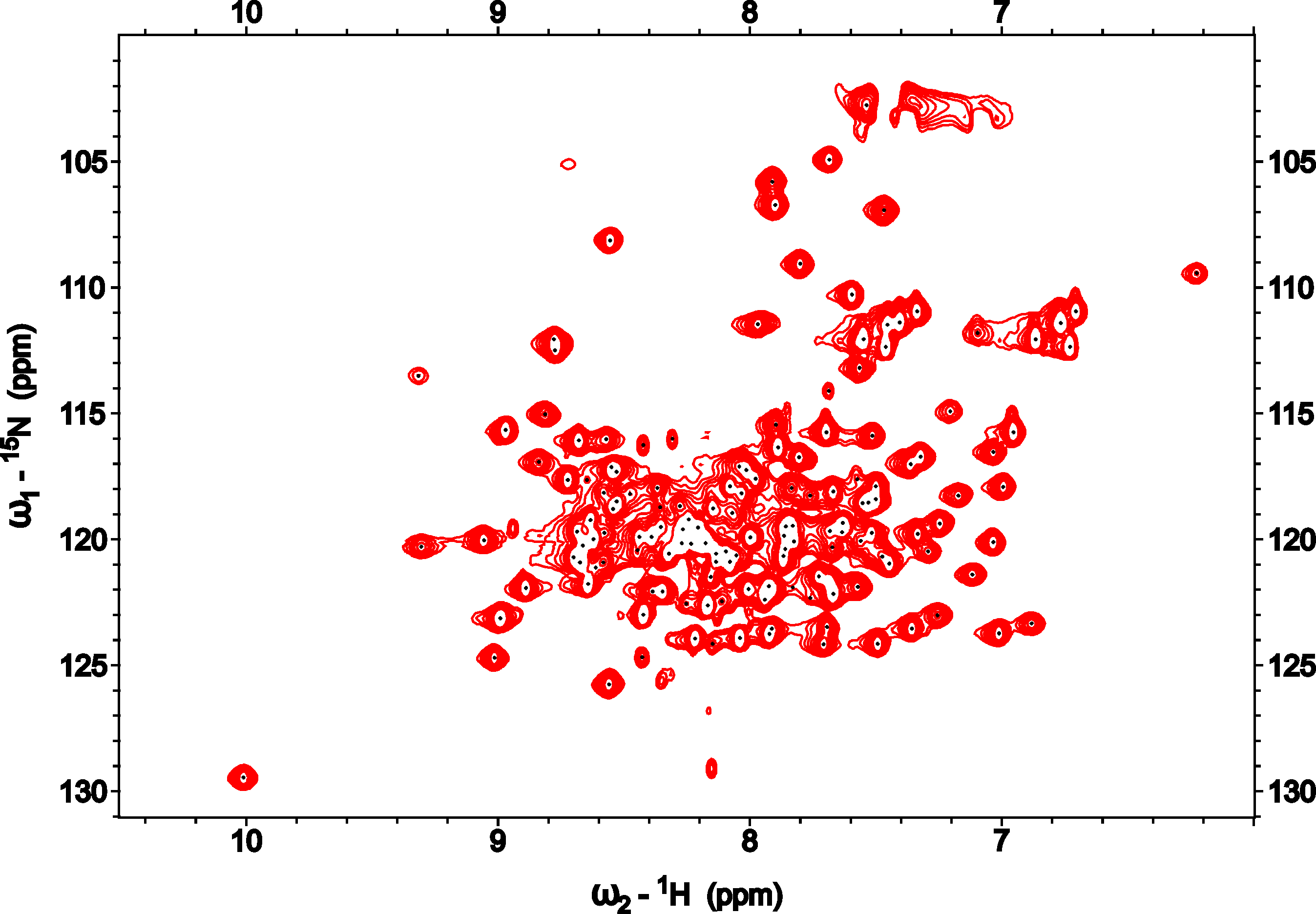

Supplement: Figure S1 — Characterization of Ani s 5 by analytical ultracentrifugation. Distribution of sedimentation coefficients measured at the NMR conditions. (TIF) [file pntd.0002735.s001.tif]

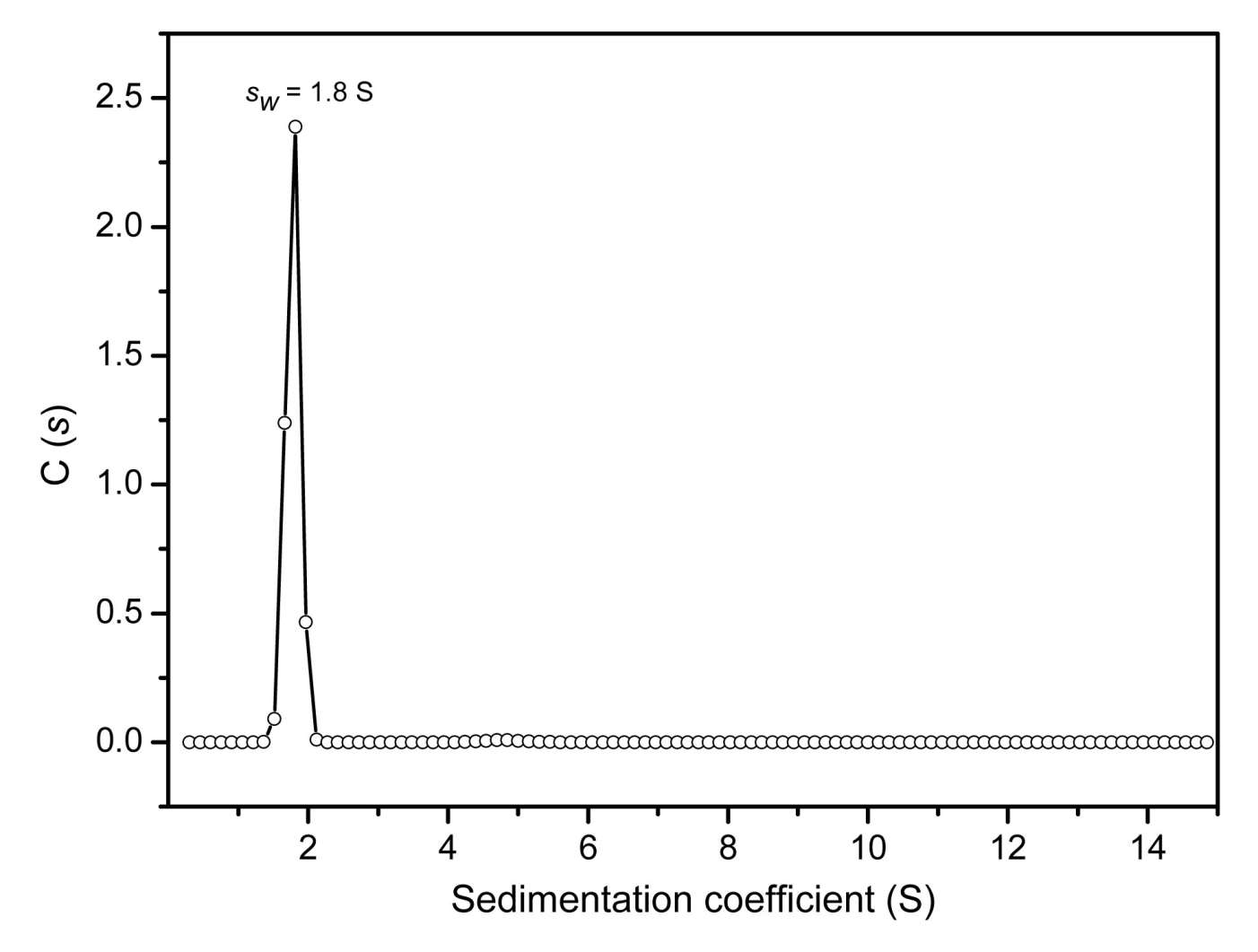

Supplement: Figure S2 — 1H-15N HSQC spectrum of Ani s 5 recorded in a 800 MHz Bruker spectrometer at pH 3.5 and 35°C. Extensive signal overlap can be observed in the region 1H: 8.7–8.0 ppm, 15N: 122–117 ppm. (TIF) [file pntd.0002735.s002.tif]
